# Predictive sulfur metabolism – a field in flux

**DOI:** 10.3389/fpls.2014.00646

**Published:** 2014-11-18

**Authors:** Alexander Calderwood, Richard J. Morris, Stanislav Kopriva

**Affiliations:** ^1^Department of Computational and Systems Biology, John Innes CentreNorwich, UK; ^2^Botanical Institute and Cluster of Excellence on Plant Sciences, University of Cologne, Cologne BiocenterCologne, Germany

**Keywords:** sulfur, flux, genome scale, flux balance analysis, kinetic modeling, metabolic control analysis

## Abstract

The key role of sulfur metabolites in response to biotic and abiotic stress in plants, as well as their importance in diet and health has led to a significant interest and effort in trying to understand and manipulate the production of relevant compounds. Metabolic engineering utilizes a set of theoretical tools to help rationally design modifications that enhance the production of a desired metabolite. Such approaches have proven their value in bacterial systems, however, the paucity of success stories to date in plants, suggests that challenges remain. Here, we review the most commonly used methods for understanding metabolic flux, focusing on the sulfur assimilatory pathway. We highlight known issues with both experimental and theoretical approaches, as well as presenting recent methods for integrating different modeling strategies, and progress toward an understanding of flux at the whole plant level.

## INTRODUCTION

Sulfur is an essential nutrient; available in the soil as sulfate, plants are able to reduce inorganic sulfur, for use in a large number of primary and secondary metabolites.

Unsurprisingly, the study of reductive sulfur assimilation by plants is often pragmatically motivated; *Brassicaceae* especially have large sulfur requirements, and the quality and yield of oilseed rape is known to be affected by low sulfur availability (De Pascale et al., 2008). Furthermore, the importance of sulfur metabolites in diet and health (Sekiz et al., 1975; Tawfiq et al., 1995; Tripathi and Mishra, 2007; Traka and Mithen, 2011), their intrinsic economic value (Li et al., 2004), and conferred tolerance to abiotic or biotic stresses in the plant (Gatehouse, 2002; Bednarek et al., 2009; Yadav, 2010) has led to an interest in manipulating their production.

A tenet of metabolic engineering is that with sufficient understanding of the components, reactions, and fluxes through a pathway we can rationally design modifications that improve, for instance, the production of a desired metabolite. This interest in, and comparative lack of examples of successful sulfur pathway engineering in plants suggests that approaches thus far have failed to significantly advance our understanding of sulfur assimilation at some level.

For plants, all is flux; all biological responses are ultimately to direct the movement of molecules and energy through the metabolic network in the most appropriate way, often acting to buffer changes in metabolite levels (Mugford et al., 2011). Consequently, understanding the control of flux is a pre-requisite for successful metabolic engineering. Unfortunately, this dynamic property is comparatively difficult to measure and interpret, and thus requires the integrated involvement of theoretical biology. Mathematical modeling has developed a number of approaches to understand control of flux through metabolism, ranging from theoretical frameworks to integrate experimental results, to highly detailed kinetic models of small fragments of a pathway, to constraint-based methods which can encompass the entire reactome.

Here, we review the most commonly used methods for studying flux, focusing on the sulfur assimilatory pathway, not just because of the commercial and scientific importance of sulfur, but because it illustrates well the more general challenges and weaknesses of each approach. Starting with the difficulties of experimental attempts to partition control of flux among the enzymes of the pathway, we then consider small scale kinetic models of several pathway branches, and flux balance analysis (FBA), as well as recent approaches to integrate different modeling strategies, and progress toward an understanding of flux at the whole plant level.

### SULFUR ASSIMILATORY PATHWAY

**FIGURE 1:**
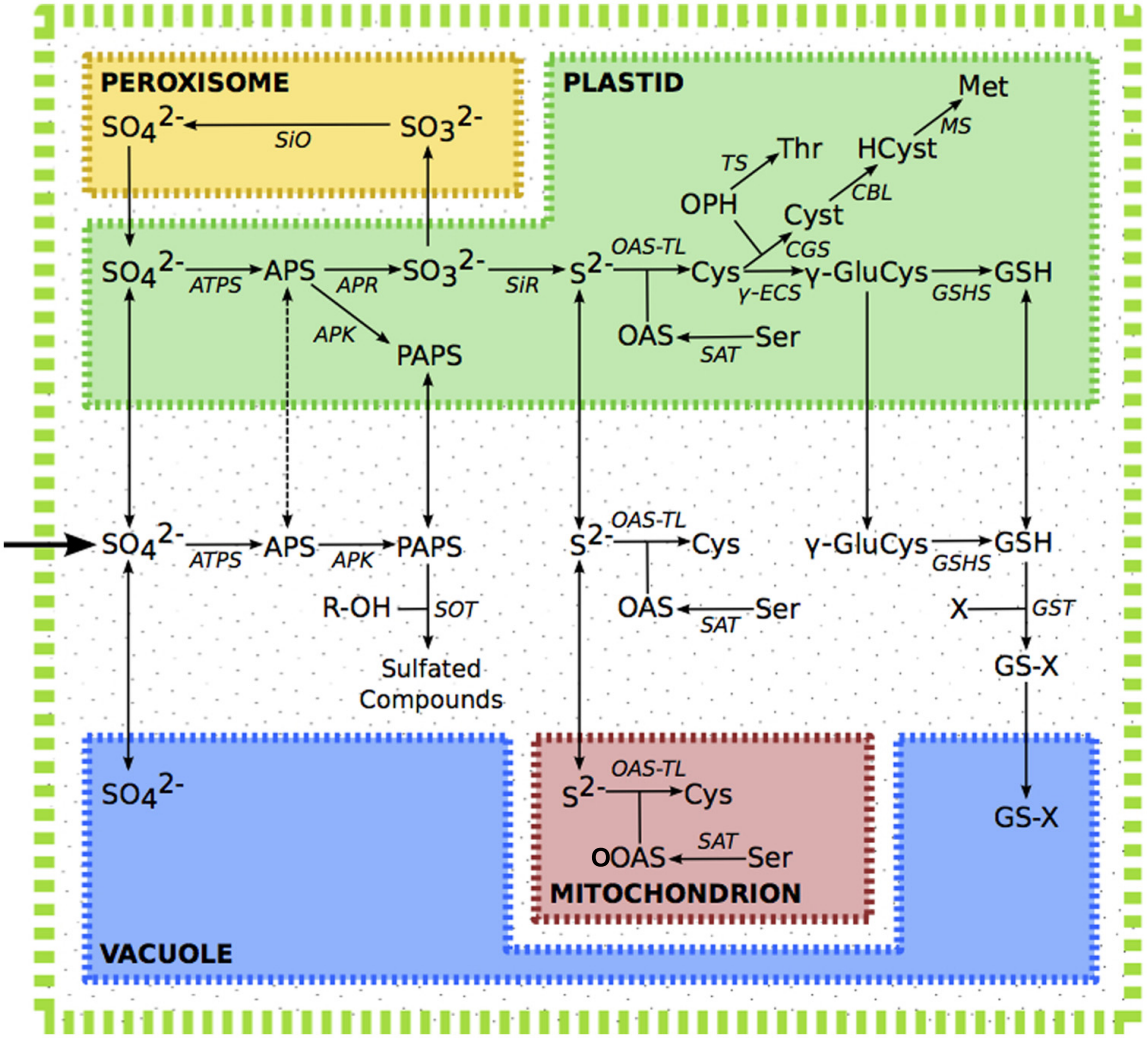
**The sulfur assimilation pathway.** Dashed line indicates putative transport of APS. Metabolite abbreviations; APS, adenosine 5′-phosphosulfate; Cys, cysteine; Cyst, cystathionine; γ-GluCys, γ-glutamyl-cysteine; GSH, glutathione; GS-X, glutathione conjugate; Hcy, homocysteine; Met, methionine; OAS, *O*-acetylserine; OPH, *O*-phosphohomoserine; PAPS, 3′-phosphoadenosine 5′-phosphosulfate; R-OH, hydroxylated precursor; Ser, serine; Thr, threonine. Enzyme abbreviations; APK, APS kinase; APR, APS reductase, ATPS, ATP sulfurylase; CBL, cystathionine β-lyase; CGS, cystathionine γ-synthase; γ-ECS, γ-glutamyl-cysteine synthetase; GSHS, glutathione synthetase; GST, glutathione-*S*-transferase; MS, methionine synthase; OAS-TL, OAS(thiol)lyase; SAT, serine acetyltransferase; SiO, sulphite oxidase; SiR, sulphite reductase; TS, threonine synthase.

The sulfur assimilatory pathway has been recently reviewed (Takahashi et al., 2011). In summary; sulfate is taken up from the environment, facilitated by specialized transporters. A large fraction of the sulfate is stored in the vacuole, while sulfate in chloroplasts or the cytosol is activated by ATP sulfurylase, forming adenosine 5′-phosphosulfate (APS). APS may then either be further phosphorylated by APS Kinase (APK) or reduced by APS Reductase (APR). Phoshorylation of APS forms 3′-phosphoadenosine 5′-phosphosulphate (PAPS), which acts as a promiscuous donor of activated sulfate, and is involved in the modification of a variety of proteins, saccharides, and secondary metabolites, including desulfo-glucosinolates. In primary assimilation, APS is instead reduced in the plastid to sulfite by APR, and then to sulfide by sulfite reductase (SiR). Sulfide in chloroplasts, mitochondria and the cytosol, is incorporated into *O*-acetylserine (OAS) to form cysteine, the precursor of all organic compounds containing reduced sulfur. Cysteine in the plastid may be converted into glutathione (GSH) via γ-glutamyl-cysteine, or reacts with phosphohomoserine, to form cystathionine, which can then be converted to methionine via homocysteine. Excess sulfite can be oxidized in the peroxisome back to sulfate by sulfite oxidase (**Figure 1**).

## SULFUR FLUX CONTROL – MEASURE BY MEASURE

Efforts to experimentally characterize flux through the assimilation pathway are based on accumulation of radiolabelled ^35^S, from ^35^SO_4,_ into various metabolite pools (Koprivova et al., 2000; Vauclare et al., 2002; Mugford et al., 2011). Assuming that over the timescale considered there is no significant turnover of the most downstream metabolites measured, this allows calculation of sulfur flux from SO_4_ through the pathway. By measuring alterations to flux distribution under genetically (Khan et al., 2010; Mugford et al., 2011), and environmentally (Koprivova et al., 2000; Vauclare et al., 2002; Scheerer et al., 2010) perturbed conditions it was hoped that insights could be gained into the control of flux through the sulfur assimilation pathway, and into various organic molecules.

### CONTROL IS DISTRIBUTED

To quantify control of flux, Vauclare et al. (2002) applied the metabolic control analysis (MCA) framework (for a comprehensive introduction to MCA, see Fell, 1992). Based on flux correlation with decreased APR activity, they calculated that APR has a large proportion of the total control of flux through the assimilatory pathway. From this, and several qualitative studies (Tsakraklides et al., 2002; Loudet et al., 2007), the hypothesis arose that APR is the key enzyme, controlling flux through the reductive assimilation pathway (Vauclare et al., 2002; Yoshimoto et al., 2007; Davidian and Kopriva, 2010; Scheerer et al., 2010). Consistent with this idea, APR has been shown to be highly regulated by demand for reduced sulfur products (Lappartient et al., 1999; Kopriva, 2006; Davidian and Kopriva, 2010; Takahashi et al., 2011), internal sulfate levels (Lee et al., 2012), and other environmental signals (Jost et al., 2005; Koprivova et al., 2008; Lee et al., 2011; Huseby et al., 2013).

More recently, however, a number of different enzymes have also been implicated in altered flux through the sulfur reduction pathway; Khan et al. (2010) found that SiR knockdown plants have a strongly reduced flux to thiols, variation in ATPS has been shown to cause altered flux of sulfur into primary metabolism (Koprivova et al., 2013), and reduction in APK increased flux through primary assimilation (Mugford et al., 2011). These results suggest that flux control is more complicated than had been previously thought, and extends beyond the APR enzyme. This distribution of control among multiple enzymes is a common feature of metabolic pathways (Thomas and Fell, 1998).

### DIFFICULTY OF APPLYING MCA FRAMEWORK TO EXPERIMENTS

Distributed control of flux means that a quantitative understanding, as attempted by Vauclare et al. (2002), becomes increasingly important for successfully engineering overproduction of metabolites. However, the results of this kind of perturbation experiment, in which the activity of an enzyme is artificially increased or decreased, are difficult to interpret within the MCA framework due to regulatory interactions, and non-linear changes in control coefficients with enzyme activity.

**FIGURE 2:**
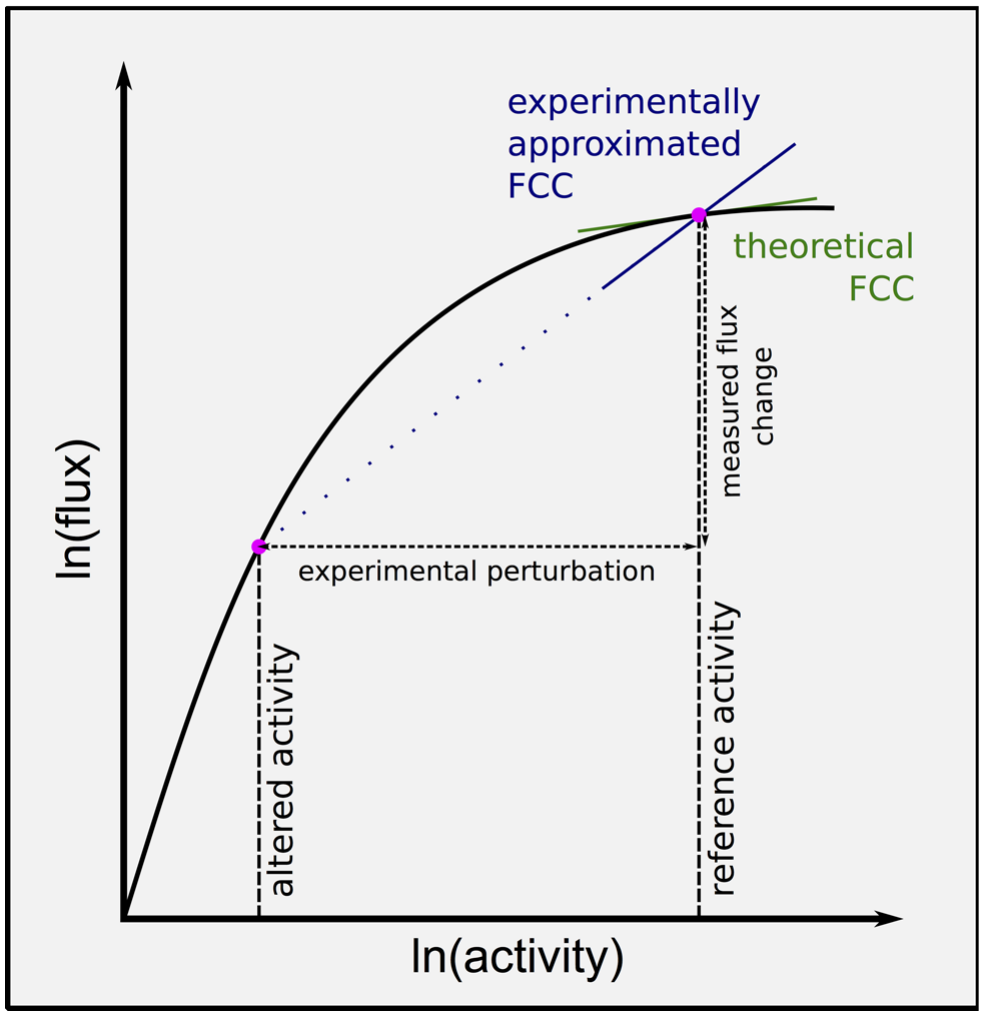
**Flux control coefficient varies non-linearly with enzyme activity, confounding experimental approximations.** Flux control coefficients (FCCs) can be computed from the gradient of the ln(flux) versus ln(activity) curve for a given enzyme. Most experimental attempts to approximate the FCC at the reference state measure the change in flux through the pathway with change in an enzyme activity. Larger experimental perturbations result in less accurate estimates, and knock down experiments can be expected to overestimate FCC at the reference state. Ideally multiple perturbations should be made, and the curve fitted to the data, allowing a point estimate of FCC at the reference state.

Metabolic control analysis defines flux control coefficients (FCCs) as the sensitivity of flux through the pathway to an infinitesimal change in a given enzyme activity from the reference state (**Figure 2**). These coefficients can be interpreted as a measure of the ‘rate limitingness’ of the enzyme to flux though the pathway, and potentially used to identify targets for overexpression to increase flux to metabolites of interest.

To determine FCCs experimentally, by measuring the effect of a genetic perturbation on flux through the pathway requires the assumption that no other enzyme activities change in compensation (Fell, 1992). However, demonstrating that this is the case is challenging (Vauclare et al., 2002; Scheerer et al., 2010), and given the complex regulation of the pathway by a number of metabolites (Takahashi et al., 2011) is generally unlikely to be true. Although for some simple purposes this may not matter, it does hinder an understanding the root causes of changes of flux through the network, limiting the applicability of any findings.

Flux control coefficients vary non-linearly with enzyme activity. Therefore although a large experimental change in enzyme activity may result in significantly altered flux through the pathway, this does not mean that the enzyme actually has a high control coefficient in the unperturbed state (**Figure 2**). Ideally several magnitudes of perturbation would be made, and used to estimate the control coefficient in unperturbed conditions, however, this has not been done to date within the sulfur community (Vauclare et al., 2002; Khan et al., 2010). Furthermore, this means that experimentally approximated control coefficients cannot be directly compared to each other, as genetic perturbations vary in magnitude (Vauclare et al., 2002; Khan et al., 2010). It is therefore still not quantitatively clear which reactions have how much control of sulfur assimilation, even under controlled experimental conditions.

This non-linearity also means that MCA is not a robust predictive framework for engineering; control coefficients at the reference state are not necessarily likely to reflect control coefficients under genetically altered conditions. Interestingly, Scheerer et al. (2010) found that distribution of FCCs through the sulfur assimilation pathway varied with environmental conditions and organism, likely due to altered enzyme expression levels. This highlights the importance of a predictive understanding of the controlling steps through the pathway, not only to more robustly predict the effect of genetic alterations, but also due to the impracticality of experimentally determining control distributions under all environments of interest.

## SMALL IS BEAUTIFUL – KINETIC MODELING

Although experimental investigations into control of flux through the pathway have been useful in qualitatively identifying important enzymes, this approach is limited, as multiple controlling enzymes and non-linear dynamics make predicting behavior away from measured conditions difficult. This was clearly seen in the analysis of poplar roots, where despite an increase in APR activity in many conditions, only few resulted in higher flux (Scheerer et al., 2010). Furthermore, the data generated experimentally is not easily integrated into a formal framework for analysis. In contrast, the MCA framework is easily applied to kinetic models of the pathway, which can be used not only to calculate control coefficients at the reference state more accurately than is possible experimentally, but also to simulate altered conditions. The difficulty lies, however, in producing an appropriate model.

Kinetic modeling of metabolic pathways is well established (see Curien et al., 2014 for a practical introduction). Models comprise a coupled system of ordinary non-linear, differential equations, functions of metabolite concentrations and kinetic parameters, which specify the rate of a reaction. A given pathway system can be mapped onto these equations, and solved numerically using a range of freely available software (Copeland et al., 2012).

In sulfur metabolism, this has allowed not only dissection of flux control distribution at several points (Curien et al., 2003; Mendoza-Cózatl and Moreno-Sánchez, 2006), but also predictions about how environmental perturbation changes control of flux to GSH (Mendoza-Cózatl and Moreno-Sánchez, 2006), and suggested engineering interventions to modify levels of sulfur metabolites. For example Curien et al. (2003) were able to predict that overexpressing cystathionine-γ-synthase would allow overproduction of methionine, without compromising threonine production, and that this was therefore a better strategy than knocking down threonine synthase (TS).

However, although kinetic models have yielded useful insights into flux through the sulfur assimilation pathway, the rarity of models published in this area hints at the difficulties of the approach.

### PROBLEMS WITH KINETIC MODELS

Kinetic models require detailed understanding of the biological pathway under study, at the structural, thermodynamic, and kinetic levels. In many instances the pathway structure is well known, and thermodynamic data are either available (Goldberg et al., 2004), or can be calculated approximately (Jankowski et al., 2008), however, incomplete knowledge of enzyme kinetic parameters remains as the biggest hurdle to model building, particularly given that isoenzymes in different tissues or compartments often display different kinetics. Strategies for determining parameter values can be broadly split into measurement, and estimation approaches.

#### Parameter measurement

For small models, it may be possible to measure all kinetic parameters required. Curien et al. (2003) were able to measure the kinetic parameters of TS and cystathionine-γ-synthase for their model of the branch point of methionine and threonine biosynthesis *in vitro*, however, the large experimental effort required (Stitt and Gibon, 2014; Tummler et al., 2014; van Eunen and Bakker, 2014) makes this a comparatively rare example; it is more common to search the literature to recover the majority of parameters required (Rohwer, 2014). Several databases (Schomburg et al., 2004; Wittig et al., 2012) facilitate the search for previously determined kinetic parameters, however, generally poor coverage, particularly for allosteric regulation, means that it is accepted practice to use whichever parameters are available, either from experiments under differing conditions, or from orthologous proteins (Rohwer, 2014). The validity of transferring parameters in this way is generally unclear (Stitt and Gibon, 2014), the exception being enzyme activity parameters, which are acknowledged to vary so greatly with environment, that they should be measured under the condition of interest (Curien et al., 2014). There has been some speculation that advances in robotics, and microfluidics could lead to ‘omics style investigations’ into enzyme kinetics (Gibon et al., 2004; Sjostrom et al., 2013), however, a reliable high throughput pipeline has not to our knowledge been developed, and poor coverage is likely to remain a problem in the immediate future.

Kinetics databases highlight a further shortcoming of kinetic parameters measured *in vitro*; the assay conditions used are typically far from the *in vivo* environment seen by the enzyme. This problem of non-physiological *in vitro* assay media can be seen in Curien et al. (2003), where the use of high phosphohomoserine media, likely contributed to a poor initial model fit to data. Initiatives to design more *in vivo* like *in vitro* media, are underway for several microorganisms (Garcia-Contreras et al., 2012; Goel et al., 2012; Leroux et al., 2013), but to the best of our knowledge, no such effort has been reported in plants, where the problem is exacerbated by the presence of multiple subcellular compartments, each with a unique environment. In the sulfur assimilation pathway, three out of the five reactions converting sulfate to cysteine occur in multiple compartments (Takahashi et al., 2011) and thus likely require multiple sets of kinetic parameters.

#### Parameter estimation

The difficulties of obtaining experimentally measured kinetic parameters mean that in the vast majority of published models, at least some parameters are fitted by minimizing the difference between model predictions (e.g. of flux through the path), and experimental measurements (Tummler et al., 2014). Aside from the experimental difficulties of acquiring data, especially within subcellular compartments, one problem with this approach is overfitting; assigning parameter values to fit the data more precisely than is justified. As a result, many models parameterized using a top down approach lose predictive accuracy as conditions move away from those at which the parameters were fitted (Hawkins, 2004).

A number of approaches have been developed based on sensitivity of model predictions to parameter values to analyze parameter identifiability, and calculate confidence intervals for parameters and predictions (Cotten and Reed, 2013; Kravaris et al., 2013). However, these problems have not always been rigorously considered in the literature. Mendoza-Cózatl and Moreno-Sánchez (2006) ignored possible interaction terms between parameters in their sensitivity analysis, and Curien et al. (2003) provide no indication of the robustness of their predictions to error in measured parameter values.

#### Parameter reduction

Given the problem of estimating a large number of unknown parameters with limited data, most models tend to use lumped, empirical rate laws, which aim to capture the salient kinetic features, whilst minimizing the number of parameters required, rather than complicated, mechanistic laws (Heijnen, 2005; Curien et al., 2014; Rohwer, 2014). However, although this simplification can be useful (Costa et al., 2011), it inevitably leads to a loss in model fidelity. Curien et al. (2003) found that even replacing a ping-pong rate law with relatively complex Michaelis–Menten kinetics led to their model losing the experimentally identified insensitivity to cysteine concentration of cystathionine γ-synthase.

### SMALL MAY NOT BE SUFFICIENT

A minimal model of a subsystem should include everything that affects the internal variables of the model (Curien et al., 2014), however, in practice, lack of biological knowledge can make it difficult to know what has to be included. Within the sulfur assimilation pathway, it is still often unclear which metabolites regulate enzyme activity allosterically, although it seems that many potentially can (Vauclare et al., 2002; Hopkins et al., 2005; Rouached et al., 2009; Hubberten et al., 2012; Lee et al., 2012). This means that, for example, even a small model of the APR, APK branch point must be large enough to consider the reduction pathway at least as far as GSH production, as this feeds back to regulate APR (Vauclare et al., 2002; Hacham et al., 2014) and possibly APK through changes in redox environment (Ravilious et al., 2012). This results in the requirement for a large number of kinetic parameters.

The extent to which models can be simplified and still remain useful is unclear, as an overly reduced model system can result in inaccurate predictions. For example ignoring phosphohomoserine production meant that Curien et al. (2003) were only able to identify a subset of the intervention steps that have since been experimentally shown to increase methionine production Lee et al. (2005) and Mendoza-Cózatl and Moreno-Sánchez (2006) demonstrated the importance of considering demand for GSH, as well as its production by finding that including demand results in large changes in the control coefficients of synthesizing reactions.

It is possible that a much larger metabolic network has to be considered when modeling sulfur assimilation than just the pathway itself, for example, GSH production at night is limited by availability of glycine, as its major source is photorespiration (Noctor et al., 1999). Integration of sulfur assimilation within the wider metabolic network is demonstrated by the tight coordination of sulfur uptake with nitrogen and carbon availability (Koprivova et al., 2000; Kopriva and Rennenberg, 2004; Nero et al., 2009), and the broad range of conditions which have been shown to alter enzyme activities in the pathway (Kopriva et al., 1999; Koprivova et al., 2008; Huseby et al., 2013). For instance cysteine links both nitrogen and carbon metabolism to sulfur assimilation via OAS. OAS availability is likely a dominant factor in regulating the production of cysteine by controlling formation of the cysteine synthase complex (Birke et al., 2012), and so its availability has to be considered in models of cysteine synthesis. Furthermore, as at least under some conditions, cysteine availability limits production of downstream metabolites such as methionine and GSH (Noctor et al., 1996), and these downstream metabolites can regulate upstream components (Vauclare et al., 2002; Hacham et al., 2014), therefore this link to wider metabolism should be acknowledged whichever part of the sulfur pathway is being studied.

### THE DIFFICULTY WITH LARGER MODELS

Unfortunately, as model size increases, the problems of unknown parameters, and rate laws become extremely difficult to overcome. To generate a large kinetic model, simplifying assumptions about parameter values (Smallbone and Mendes, 2013), and rate laws (Alves et al., 2008), are frequently made, but this often results in poor model quality away from the fitted conditions (Chakrabarti et al., 2013) and so is of limited predictive value.

Other kinetic modeling frameworks acknowledge the inherently greater unknowns of a large system, and use the available data to define a cadre of related models, or sample feasible parameter space, reflecting either structural, or parameter and rate law uncertainty in their predictions (Famili et al., 2005; Steuer et al., 2006; Tran et al., 2008; Miskovic and Hatzimanikatis, 2011). Some of these approaches have resulted in the production of large kinetic models, in the order of 200 metabolites and reactions (Khodayari et al., 2014), but do not scale well to bigger models. As model size increases, parameter space expands enormously (Zamora-Sillero et al., 2011), resulting in prohibitive computational requirements (Link et al., 2014). As such, kinetic modeling currently does not scale to the size that is likely to be required to gain a holistic understanding of flux through sulfur related pathways.

## BIGGER IS BETTER – GENOME SCALE MODELS

In contrast, constraint-based modeling provides a number of hugely scalable, largely parameter free methods for understanding flux through large metabolic networks (Lewis et al., 2012; Bordbar et al., 2014a). Here we focus on FBA as the most commonly used constraint-based method, the only method currently applicable to the genome scale, and a foundation for of many closely related variants.

Flux balance analysis (Varma and Palsson, 1994) is a powerful technique to estimate internal flux distributions within a large-scale network using only the structure of the reaction network, an objective function, and a small number of measured nutrient uptake fluxes as constraints, (for a practical introduction, to the method, see Grafahrend-Belau et al., 2014). By assuming metabolic steady state, and that fluxes are distributed so as to maximize some cellular objective, feasible flux space is reduced, and a subset of biologically likely internal flux distributions are predicted.

Flux balance analysis can be directly used in a number of areas, including understanding metabolic efficiency (Chen and Shachar-Hill, 2012), interpreting ‘omics data’ (Toepfer et al., 2013; Simons et al., 2014b), and predicting novel metabolic pathways (Hay and Schwender, 2011; Bordbar et al., 2014b). Furthermore extensions to the method interpret structural properties related to control of flux (Notebaart et al., 2008; Sajitz-Hermstein and Nikoloski, 2013), predict how flux distribution changes in response to genetic and environmental changes (Segre et al., 2002; Cheung et al., 2014), and suggest optimal intervention strategies to engineer metabolite production (Zomorrodi et al., 2012; Tomar and De, 2013; Ohno et al., 2014), as well as having a number of other applications (Papp et al., 2011; Bordbar et al., 2014a). This array of methods has been recently reviewed (Lewis et al., 2012), but continues to rapidly expand.

### PROBLEMS WITH GENOME SCALE MODELS

In spite of a profligacy of analytical methods and well-documented metabolic engineering case studies in microbes, application of FBA based methods to plants has been limited to date. This is likely due to challenges in genome scale model construction, and the assumptions of the FBA method, as will be discussed below.

#### Model construction

Despite a large number of available tools (Kim et al., 2012), construction of a genome scale model is not a facile task, and particularly in plants remains a laborious undertaking (Saha et al., 2014; Simons et al., 2014a). Here we highlight some of the difficulties biological unknowns cause in creating even single cell type models of plants.

Although primary metabolism is well understood, the generally poorer understanding of the huge plant secondary metabolism (Shachar-Hill, 2013), is reflected in the focus of models published to date (Poolman et al., 2009; Williams et al., 2010; Cheung et al., 2013). The problem of unknown metabolites was recently highlighted for sulfur metabolites in particular (Glaeser et al., 2014), and the potentially large numbers of missing reactions suggested by the large proportion of genome content with unknown function (Seaver et al., 2012) could also adversely affect prediction quality.

All published genome scale models of plant metabolism include compartmentalization to some extent, but the problem of biological unknowns again raises concern over the quality of some of the assignations. Wide variation between models in which compartments reactions occur (Poolman et al., 2009; Masakapalli et al., 2010; Mintz-Oron et al., 2012) suggests that despite databases of subcellular enzyme location (Heazlewood et al., 2007; Sun et al., 2009), and parsimony based methods for extending database coverage (Mintz-Oron et al., 2012), the number of reactions which can be confidently assigned to particular compartments, and in particular to the vacuole, is probably much lower than occur in reality (Krueger et al., 2011). This reflects the current difficulty of experimentally determining subcellular reaction location.

Additionally, transport between compartments is often poorly understood; even in well-studied parts of metabolism, it is not always clear which metabolites can move between compartments, and the energetic costs of transport reactions are rarely known. This is shown in the sulfur assimilatory pathway by the only recent identification of PAPS transport between the plastid and cytosol (Gigolashvili et al., 2012).

#### FBA analysis

***Objective functions.*** In addition to defining network structure, some biological knowledge of the system is required to choose an appropriate objective function. One commonly used objective is maximization of biomass production (Feist and Palsson, 2010), which is equivalent to finding the most efficient way of generating biomass from nutrients taken up by the cell (Zarecki et al., 2014). Although maximization of biomass is generally accepted as a good objective function for bacteria in log phase, the accuracy of fluxes predicted using it vary with environment, growth phase, and species, suggesting that this is not always appropriate (Schuster et al., 2008; Feist and Palsson, 2010).

A number of other objective functions have been considered in the literature, most often tied either explicitly or implicitly to efficiency in some regard (Chen and Shachar-Hill, 2012), although recently other objectives have been proposed which either aim to maximize growth rate (Zarecki et al., 2014) or minimize conflict with ‘omic data’ (Becker and Palsson, 2008; Collins et al., 2012). Much literature assessing the performance of these various different objective functions in correctly predicting observed growth, gene essentiality or flux states in bacteria (Burgard and Maranas, 2003; Schuetz et al., 2007; Feist and Palsson, 2010), and plants (Cheung et al., 2013) has been produced, however, it is not clear that organisms act to optimize a single objective function, even under constant conditions (Nagrath et al., 2007; Schuetz et al., 2012; Harcombe et al., 2013).

Combined objectives give the most accurate predicted flux distributions, and both in bacteria (Schuetz et al., 2012), and Eukaryotes (Nagrath et al., 2007), cell fluxes apparently occupy a Pareto surface, at which several objectives trade-off against each other, as further increase in one objective leads to a decrease in another. It is not obvious how this problem of competing objectives can be addressed by FBA; defining an appropriate objective function becomes much more difficult, because although frameworks for optimizing multiple objectives are well established (Oh et al., 2009; Zomorrodi and Maranas, 2012; Zomorrodi et al., 2014), the lack of a predictive understanding of which trade-offs are likely to apply, limits their application, as it is likely to vary with species, environment, and developmental state.

***Degeneracy.*** Another problem is degeneracy, FBA is often unable to distinguish between a number of flux distributions, which all maximize the objective function. Although this degeneracy of predicted distributions is often considered undesirable (Pozo et al., 2014), it is in fact likely to reflect biological reality. Degenerate optimal solutions are consistent with robustness, which seems to be a common feature of biological networks (Kitano, 2004), and a population of cells is unlikely to be adequately described by a single flux distribution (Labhsetwar et al., 2013).

The real difficulty associated with degenerate flux distributions is that experimentally measured fluxes in bacteria often actually exist in suboptimal regions, which allow large flux variation (Schuetz et al., 2012; Harcombe et al., 2013; Roman et al., 2014) without further compromising the best combination of assumed objectives. Although the extent to which apparent sub-optimal distributions arise through the averaging of measured fluxes in a heterogeneous population, rather than sub-optimality in a single cell is unclear, the FBA assumption that flux is distributed in order to maximize an objective function may only be a useful approximation in specific cases. FBA based methods are beginning to appear that address the need to consider only partially optimized distributions (Wintermute et al., 2013), but sub-optimal distributions are a major challenge for the FBA framework, which given the sophistication and size of plant metabolic networks, and numerous differentiated cell types, is likely to be particularly relevant to their study.

In spite of these difficulties, an increasing number of studies have accurately predicted flux distributions in plants cells using FBA (Dal’Molin et al., 2010a; Williams et al., 2010; Hay and Schwender, 2011; Saha et al., 2011; Cheung et al., 2013). Precise external flux measurements have been shown to be more important for accurately predicting internal fluxes than the objective function used (Cheung et al., 2013), and although current approaches to use transcriptomics data to improve flux prediction accuracy have been recently questioned (Machado and Herrgard, 2014), fluxomics data generated by metabolic flux analysis [recently reviewed from a sulfur perspective by Rennenberg and Herschbach (2014)] can be used to add additional constraints, and further improve prediction accuracy (Hay and Schwender, 2011). It seems only a matter of time before FBA is used to facilitate engineering outcomes in plant cell cultures.

## SULFUR AND BEYOND – TOWARD WHOLE PLANT FLUX MODELS

There is great interest in bridging the gap between the long tradition of eco-physiological agronomic models (Keurentjes et al., 2013), and molecular models. Combined, these two approaches could provide an integrated understanding of control of economically important traits (Baldazzi et al., 2012; Poorter et al., 2013). This fusion requires the ability to model differentiated tissue, at least at the organ level, and consider dynamic changes to flux.

Genome scale plant models to date have generally focused on cell cultures grown in suspension (Williams et al., 2010; Cheung et al., 2013) and so bypassed the problem of differentiation, but a particular challenge in whole plant models is the large number of cell types present. Although attempts to address this remain fairly crude, and restricted to models of only a few cell types or organs, the framework, in which proteomics data is integrated into constraint-based models to generate tissue, or organ specific sub-models (Mintz-Oron et al., 2012), which can then be coupled together, and used to predict fluxes through heterogeneous material (Dal’Molin et al., 2010b; Grafahrend-Belau et al., 2013) is established.

The greatest limitation of the FBA method is that it can only consider a steady state snapshot of flux distribution, especially as the extent to which metabolism at the whole plant level is ever in steady state remains unclear. Although recent work in plants has studied responses to light–dark cycles using a purely FBA approach (Cheung et al., 2014), and FBA metrics have been identified which correlate with metabolite concentration dynamics (Reznik et al., 2013), the application of a purely FBA based methodology to study dynamic systems is limited. In bacteria, there has been a movement toward integrating FBA models within a kinetic model. This is used to dynamically modify the exchange reaction constraints while FBA is repeatedly performed, allowing internal flux dynamics to be approximated (Varma and Palsson, 1994; Mahadevan et al., 2002). Output from the FBA simulation may then be fed back, to modify the kinetic model, or not (Feng et al., 2012). This hybrid modeling approach allows prediction of genome scale fluxes over time (Vargas et al., 2011; Jouhten et al., 2012), with only a few parameters required to capture input and output fluxes, biomass prerequisites, and maintenance costs.

This dynamic flux balance analysis (dFBA) approach makes a pseudo steady state assumption, that intracellular metabolism equilibrates several orders of magnitude faster than extracellular changes. Although it is likely that the pseudo steady state assumption might not be justified in all aspects of plant metabolism, a recent extension to the method potentially relaxes this requirement (Birch et al., 2014). Grafahrend-Belau et al. (2013) recently used dFBA to link a multi organ model of barley metabolism with an agronomic model, and provide insight into the dynamic interaction of source and sink organs in relation to senescence. Although fairly unsophisticated in the models used and interaction framework considered, the first steps toward a whole plant model have been taken, and dFBA is likely to find wider application in the coming years.

## CONCLUDING REMARKS

Flux is perhaps the most important metric to determine for a practical, applied understanding of plant biology. Through the study of sulfur metabolism we have seen that to understand flux requires a fusion of experimental and modeling approaches, but that to date, no integration of the two satisfactorily solves the problem to provide an accurate predictive framework. Each approach considered continues to advance independently both theoretically and experimentally, but currently perhaps most promising is the joining of kinetic and constraint-based approaches, which although an immature field, has the potential to finally deliver a useful facet of the famous ‘virtual plant.’

## Conflict of Interest Statement

The authors declare that the research was conducted in the absence of any commercial or financial relationships that could be construed as a potential conflict of interest.
